# Piloting an HIV self-test kit voucher program to raise serostatus awareness of high-risk African Americans, Los Angeles

**DOI:** 10.1186/1471-2458-14-1226

**Published:** 2014-11-26

**Authors:** Robert W Marlin, Sean D Young, Claire C Bristow, Greg Wilson, Jeffrey Rodriguez, Jose Ortiz, Rhea Mathew, Jeffrey D Klausner

**Affiliations:** Department of Medicine, David Geffen School of Medicine at the University of California, Los Angeles, Le Conte Avenue, Los Angeles, CA USA; Department of Family Medicine, David Geffen School of Medicine at the University of California, Los Angeles, Le Conte Avenue, Los Angeles, CA USA; Reach LA, E Olympic Boulevard, Los Angeles, CA USA; LA Gay & Lesbian Center, Schrader Boulevard, Los Angeles, CA USA; University of California, Los Angeles, Hilgard Avenue, Los Angeles, CA USA

**Keywords:** HIV, Voucher, African American, Los Angeles, Self-testing, In-home testing, Feasibility

## Abstract

**Background:**

Up to half of all new HIV cases in Los Angeles may be caused by the 20-30% of men who have sex with men (MSM) with unrecognized HIV infection. Racial/ethnic minority MSM are at particularly high risk for being sero-unaware and due to stigma and poor healthcare access might benefit from novel private, self-testing methods, such as the recently FDA-approved OraQuick® In-Home HIV Test.

**Methods:**

From July-November 2013, we undertook a pilot study to examine the feasibility of a voucher program for free OraQuick® tests targeting African American MSM in Los Angeles. We determined feasibility based on: (1) the establishment of a voucher redemption and third-party payment system, (2) the willingness of community-based organizations (CBOs) to disseminate vouchers, and (3) the collection of user demographics, test and linkage-to-care results with an anonymous telephone survey.

**Results:**

We partnered with Walgreens® to create a voucher and third-party reimbursement system for free OraQuick® tests. Voucher distribution was divided into two periods. In total, 641 vouchers were supplied to CBOs: 274 (42.7%) went to clients and of those 53 (19.3%) were redeemed. Fifty (18.2%) of the 274 clients were surveyed: 44 (88%) were African American, 39 (78%) reported being likely to repeat voucher use, 44 (88%) reported reviewing pre-test information, and 37 (74%) the post-test information. Three (6%) of 50 survey respondents reported newly testing HIV-positive of whom all (100%) reported seeking medical care. Two withheld their results, both of whom also sought medical care.

**Conclusions:**

Developing and partnering with a commercial pharmacy to institute a voucher system to facilitate HIV self-testing with linkage-to-care was feasible. Our findings suggest the voucher program was associated with increasing the identification of new cases of HIV infection with high rates of linkage to care. Expanded research and evaluation of voucher programs for HIV self-test kits among high-risk groups is warranted.

## Background

The burden of HIV infection is particularly high among men who have sex with men (MSM) in Los Angeles (LA) County. In 2011, there were 36,330 MSM living with HIV/AIDS and in LA County, 10,833 of which were unaware of their infection [1]. That population disproportionately includes African Americans, who are the most vulnerable demographic group affected by HIV infection. In 2011, African Americans had the highest rate of infections for any demographic group at 966 per 100,000 persons in LA [2]. In addition, African American MSM in LA are 4 times more likely than white MSM not to know they are infected with HIV [3].

A recent study examined HIV testing preferences among high risk MSM in LA and found that, of 75 MSM surveyed, an in-home, immediate, and free HIV test had the highest acceptability [4]. In 2012, the FDA approved the OraQuick® In-Home HIV test, which allows for private, rapid self-testing at home, and helps to overcome stigma, which is a major barrier to testing. Stigma towards HIV infection is particularly high in the African American MSM community [5, 6], and research has shown that stigma reduces people’s willingness to test for diseases such as HIV/AIDS [5–7]. New in-home HIV testing methods may further reduce barriers due to stigma that are associated with conventional provider-based testing by making the testing experience private and self-controlled [8]. We examined the feasibility of piloting a commercial voucher program for free OraQuick® In-Home HIV Test kits targeting high-risk African American MSM in LA.

## Methods

We determined feasibility of our pilot program based on the ability to: (1) establish a functional commercial voucher redemption and third-party payment system, (2) use community-based organizations (CBOs) to disseminate vouchers, and (3) collect and analyze data from an anonymous telephone survey on user demographics, sexual behavior, prior testing practices, self-testing experience, results disclosure and linkage-to-care. Due to the very low cost of printing paper vouchers, we supplied a large number of vouchers to CBOs for a broad reaching campaign.

We partnered with three local CBOs servicing African American MSM to distribute vouchers. We created double-sided color vouchers for a free OraQuick® In-Home HIV test, each costing < $1 to print. Each voucher had a unique number that allowed us to track where it was distributed and where and when it was redeemed. The vouchers were redeemable at 12 local Walgreens, a US-based pharmacy chain, using a third party payment system. On a monthly basis, Walgreens invoiced our program at UCLA for payment based on the negotiated cost and number of redeemed vouchers. We supplied 237 vouchers to distributors in July 2013 during our first test period. During a second test period from August through December 2013, we supplied 404 vouchers with an attached survey recruitment flyer that invited participants to contact us by telephone.

Eligible survey participants had to be over 18 years of age and have received a voucher from the second test period. After obtaining informed consent, the interviewer collected participant demographic information, HIV testing history, sexual history, test result, linkage-to-care outcome and experience with the voucher program. At the conclusion of the interview, the participant was compensated with a $75 gift card.

Survey data were encoded using SurveyMonkey and descriptive frequencies were analyzed with Microsoft Excel® and STATA® 13 (StataCorp, College Station, TX). The UCLA institutional review board approved all aspects of the project (IRB#13-000790).

## Results

### Voucher dissemination

Distributors confirmed that 62 of 237 (26.2%) vouchers supplied during the first test period in July 2013 were distributed. Ten (16.1%) of 62 distributed vouchers were redeemed. During the second test period from August through December 2013, 230 of 404 (56.9%) vouchers were distributed. Forty-three (18.7%) of the distributed vouchers were redeemed. Fifty of 230 voucher recipients (21.7%) responded to our attached survey recruitment flyers.

CBOs employed different strategies in distributing their supplied vouchers. One CBO supplied a total of 144 vouchers during both phases, of which 144 (100%) were distributed and 34 were redeemed (23.6%). Vouchers were distributed at the CBO during community meetings and events, usually after a group discussion on self-testing. A second CBO used a similar strategy, distributing 25 of their 100 vouchers (25%) during both phases, 9 of which were redeemed (9%). The third CBO was supplied 250 vouchers during both phases but only distributed 11 (4.4%), of which none were redeemed. Their vouchers were given to those passing by various mobile outreach vans in Los Angeles. An additional 147 were supplied to student volunteers while exploring alternative distribution strategies, 35 of which were distributed (24.3%) and 10 of which (6.8%) were redeemed. All CBOs and volunteers were asked to target their distribution toward African American MSM but to distribute vouchers to any who were interested.

### Survey results

Survey respondents (n = 50) were young (90% under 35 years of age), primarily African American (88%), and a majority MSM (66%) (Table 1). Forty-nine of 50 survey respondents (98%) redeemed their voucher and used the HIV in-home self-test kit. Three (6.1%) of 49 reported a new positive test result and being linked to care, and an additional 2 (4.1%) did not disclose their test result but reported attending follow-up medical care. The 1 respondent who did not redeem their voucher was not asked about their test result or activities before and after taking the test, so n = 49 was used to calculate descriptive statistics. For all other survey items there were no missing data and n = 50.

**Figure 1:**
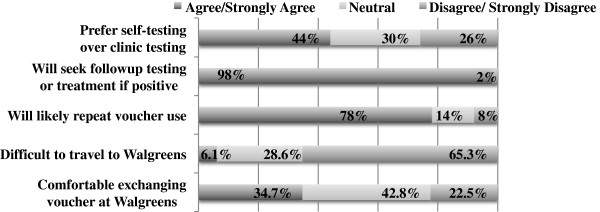
**Opinions in HIV in-home self-test voucher use survey attitudes (N = 50), Los Angeles, 2013.**

Using a Likert scale, 78% of participants reported that they were likely or very likely to use a voucher again, 65% reported that it was easy to travel to a Walgreens to redeem their voucher and 44% preferred self-testing over clinic based testing (26%) (Figure 1). About 22% of participants were uncomfortable or very uncomfortable with the in-store redemption process. One participant noted that the Walgreens staff at the store they visited was confused about the voucher, had to involve the store manager, took longer than expected, and overall the in-store process made the participant feel uncomfortable.

**Table 1:** **HIV in-home self-test voucher use survey participant characteristics Los Angeles, 2013**

Survey response	Total (n=50)
Age (years):	
**18-25**	19 *(38%)*
**26-35**	26 *(52%)*
**36+**	4 *(8%)*
Race:	
**White**	4 *(8%)*
**Black**	44 *(88%)*
**Other**	3 *(6%)*
Sexual Behavior and Gender Identification:	
**Men who have sex with women**	1 *(2%)*
**Women who have sex with men**	9 *(18%)*
**Men who have sex with men**	33 *(66%)*
**Women who have sex with women**	2 *(4%)*
**Transwomen**	5 *(10%)*
Number of New Sex Partners, Past 12 Months:	
**0 to 1**	17 *(34%)*
**2 to 3**	6 *(12%)*
**3 to 4**	13 *(26%)*
**5+**	14 *(28%)*
Condom Use:	
**Every time**	13 *(26%)*
**Frequently or usually**	29 *(48%)*
**Sometimes or less**	8 *(16%)*
Last HIV Test:	
**3 months or less**	10 *(20%)*
**3-6 months**	7 *(14%)*
**6-9 months**	10 *(20%)*
**9-12 months**	8 *(16%)*
**12+ months**	15 *(30%)*
Voucher Redemptions:	
**Redeemed**	49 *(98%)*
**Did not redeem**	1 (*2%)*
**Survey Response**	
**For Voucher Redeemers**	**Total (n=49)**
Reported Test Result:	
**Positive**	3* *(6.1%)*
**Negative**	44 *(89.8%)*
**Not disclosed**	2** *(4.1%)*
Activities Before Taking the Test:	
**Read product information or instructions**	44 *(89.9%)*
Activities After Taking the Test:	
**Read product information or instructions**	37 *(75.5%)*

*All 3 reported linkage to care.

**Both reported linkage to care.

## Discussion and conclusion

We piloted an HIV self-test voucher distribution and redemption program for free self-test kits in partnership with a large commercial pharmacy. The cost of the actual voucher was low and the major cost to the program was incurred when a voucher was redeemed. Among the sample of those surveyed who redeemed vouchers, there was a high proportion of newly identified cases of HIV infection. All newly identified cases reported linkage to care. Participants endorsed the voucher system as a means to reduce stigma associated with HIV testing through their qualitative and quantitative feedback.

We were able to track voucher use from the time we supplied them to the point when clients redeemed them, validating the functionality of our system. Many CBOs were also willing to distribute a large number of vouchers to African American MSM. Thus, we found it feasible to develop a commercial voucher system with 3rd-party reimbursement to promote HIV self-testing among high-risk African American MSM in Los Angeles.

CBOs that distributed vouchers through their membership tended to have higher distribution and redemption rates than those who solicited those passing by. In addition, distribution and redemption increased for these CBOs during the second phase due to increasing utilization of membership involvement over time. The voucher program could be sustainably used to increase the uptake of HIV self-tests by implementing permanent 3rd-party voucher reimbursement and encouraging CBOs to distribute vouchers to high-risk persons.

Our findings suggest a high acceptability for in-home testing among high-risk African American MSM, which is consistent with studies of MSM testing preferences [4, 9]. A recent survey examined the hypothetical acceptability of testing at a physician’s office, individual voluntary counseling and testing, couples’ HIV counseling and testing, expedited/express testing, rapid home self-testing using an oral fluid test, and home dried blood spot specimen self-collection for laboratory testing [9]. Home self-testing and physician’s office testing had the highest acceptability across all demographic and behavioral groups [9]. However, participants typically identified multiple testing scenarios as highly acceptable, indicating a comprehensive strategy that provides multiple testing options to the community may have the greatest effect on this population [9].

A mathematical modeling study by Katz et al. has demonstrated that a complete replacement of clinic-based testing with in-home testing amongst MSM in Seattle may result in an increased HIV prevalence [10]. However, that model doesn’t account for in-home testing being offered as a supplement to clinic-based testing, which Katz et al [10] and a recent editorial [11] have acknowledged may reduce HIV prevalence. In addition, promoting in-home testing towards groups who are untested for HIV would decrease HIV prevalence [10, 11]. Programs utilizing vouchers to promote in-home testing as a supplement to clinic-based testing can be used to evaluate these assertions, but determination of the effectiveness of such programs to decrease HIV prevalence among African American MSM will require rigorous evaluation on a larger scale.

Our pilot project had several limitations. Firstly, there were only 43 vouchers redeemed at Walgreens but 49 respondents reported redeeming a voucher. This could be due to individuals completing more than one survey or individuals incorrectly reporting their voucher redemption. Second, given there were 230 vouchers with survey recruitment materials and 49 of 50 respondents reported redeeming the voucher, there is a lack of data on those who did not redeem their voucher. Future projects should attempt to verify the uniqueness of each survey participant and collect information from non-redeemers. In addition, Walgreens stores occasionally ran out of self-test kits during the evaluation period and awareness about the program among the Walgreens staff was inconsistent. However, our ability to identify those limitations indicates the success of collecting process data for quality improvement necessary to enhance the pilot program. Lastly, our survey involved a relatively small sample size of 50, but we believed this was sufficient to assess the acceptability of participation and provide formative information on the structure of the voucher system.

A pilot study by Young et al. has found distributing HIV self-testing kits through smart vending machines to be feasible [12]. Our team plans to compare multiple methods of increasing the availability of HIV self-test kits such as the use of smart vending machines or the US mail and compare those with referrals to conventional site-based testing to find the best ways to increase HIV testing and community-level HIV serostatus awareness among high-risk groups. Continued innovation is urgently needed to address the large number of persons unaware of their HIV infection.

## Abbreviations

CBO: Community-Based Organization
LA: Los Angeles
MSM: Men who have sex with men.
